# Layers of Genius: The Mohs Method and Its Maverick Inventor

**DOI:** 10.7759/cureus.73377

**Published:** 2024-11-10

**Authors:** Michael J Brennan, Travis S Dowdle, Richard F Wagner

**Affiliations:** 1 Dermatology, Wayne State University School of Medicine, Dearborn, USA; 2 Dermatology, University of Texas Medical Branch at Galveston, Galveston, USA

**Keywords:** basal cell carcinoma, cancer surgery techniques, cutaneous oncology, dermatologic surgery, frederic e. mohs, microscopic margin control, mohs micrographic surgery (mms), skin cancer treatment, squamous cell carcinoma, tissue-sparing surgery

## Abstract

Dr. Frederic E. Mohs significantly advanced the treatment of skin cancer with the development of Mohs micrographic surgery (MMS). His career was dedicated to improving patient outcomes through innovative surgical techniques. MMS involves the precise removal of skin cancer in a stepwise manner, with each layer examined microscopically to ensure the complete removal of cancerous cells. This method has become the preferred approach for treating complex and recurrent skin cancers, particularly in areas such as the face, ears, and hands, due to its tissue-sparing precision. The procedure’s accuracy has led to high cure rates and low recurrence, solidifying its role in modern dermatologic oncology. This paper examines Mohs' life, the evolution of MMS, and its lasting influence on dermatologic surgery, addressing both the challenges and advancements within the field.

## Introduction and background

Dr. Frederic E. Mohs, often called the "Father of Mohs Surgery," is a monumental figure in dermatology whose innovative approach to skin cancer treatment revolutionized medical practice. His development of Mohs Micrographic Surgery (MMS) marked a paradigm shift in treating complex and recurrent skin cancers, offering unprecedented precision and efficacy. In the early 20th century, skin cancer treatment options, particularly for basal cell carcinoma and squamous cell carcinoma, were limited. Standard surgical procedures often result in significant tissue loss, scarring, and lower cure rates. Recognizing these limitations, Mohs dedicated his life to advancing skin cancer treatment, seeking a method that could provide precision in cancer removal while conserving as much healthy tissue as possible. His groundbreaking approach not only offered a higher cure rate compared to traditional surgical methods but also reshaped the principles of surgical oncology by making skin cancer surgery less invasive and more effective [1,2]. This introduction sets the stage for understanding Mohs' contributions by exploring his background, the medical landscape of his time, and the necessity for his innovation, ultimately laying the groundwork for an in-depth exploration of his enduring impact on dermatologic surgery.

Early life and education

**Figure 1:**
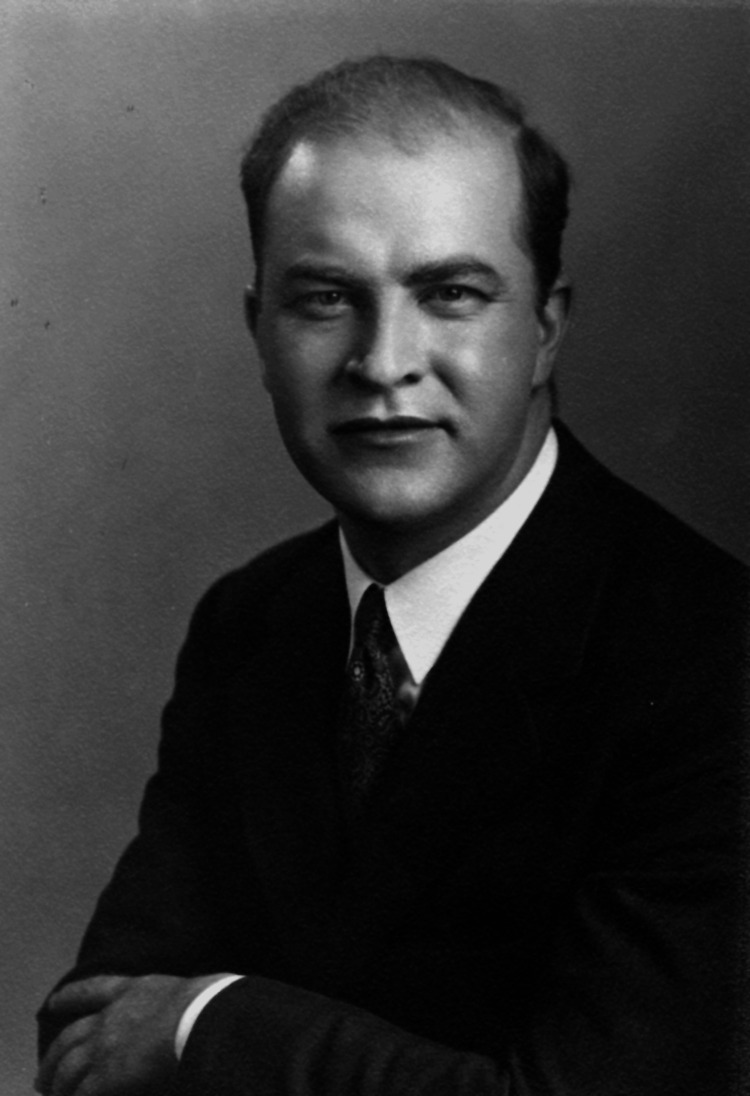
Young Dr. Frederic E. Mohs, MD Permission obtained from the University of Wisconsin Madison Archives and Record Management [4].

Born on March 1, 1910, in Burlington, Wisconsin, Frederic Mohs showed an early interest in science and medicine, an interest that would become a driving force in his career. His educational journey began at the University of Wisconsin, where he pursued a pre-medical curriculum. Mohs' academic rigor and passion for understanding complex biological processes led him to complete his medical degree from 1929 to 1934 (Figure 1) [3]. During this period, he was profoundly influenced by his mentor, Dr. Michael Guyer, a zoology professor known for his expertise in tissue preparation for microscopic examination. Under Guyer's guidance, Mohs became adept at preparing frozen tissue for histological slides, a skill that would later become fundamental to his surgical technique.

Dr. Guyer, who authored a book on tissue harvesting and processing for microscopic examination, emphasized the importance of detailed observation, drawing, and documentation - principles that would become evident in Mohs' later work in dermatology. This period of study under Dr. Guyer was crucial as it laid the foundation for his future work in dermatology, particularly his interest in cancer treatment. The meticulous approach to research and tissue analysis he learned would eventually lead to his groundbreaking development of MMS, a method that combines clinical surgery with pathology.

Development of MMS

The concept of MMS originated in the 1930s when Frederic Mohs, then a 23-year-old research assistant, discovered that zinc chloride could "fix" tissue in vivo, enabling detailed histological examination [5]. While researching chemotherapeutic pharmacologic treatments on rat models, Mohs injected various chemicals, including zinc chloride. He serendipitously found that this solution could preserve skin tissue without altering its cellular architecture [2]. This unexpected discovery paved the way for a revolutionary technique in skin cancer treatment. Building on this finding, Mohs developed a cohesive paste by combining zinc chloride with stibnite and sanguinaria canadensis [5]. When applied to tissue, following curettage, the paste allowed for bloodless excision, after which frozen sections of the excised tissue could be prepared and examined microscopically [2,6-8]. This innovative combination of histological mapping and microscopic examination laid the foundation for the procedure that now bears his name.

**Figure 2:**
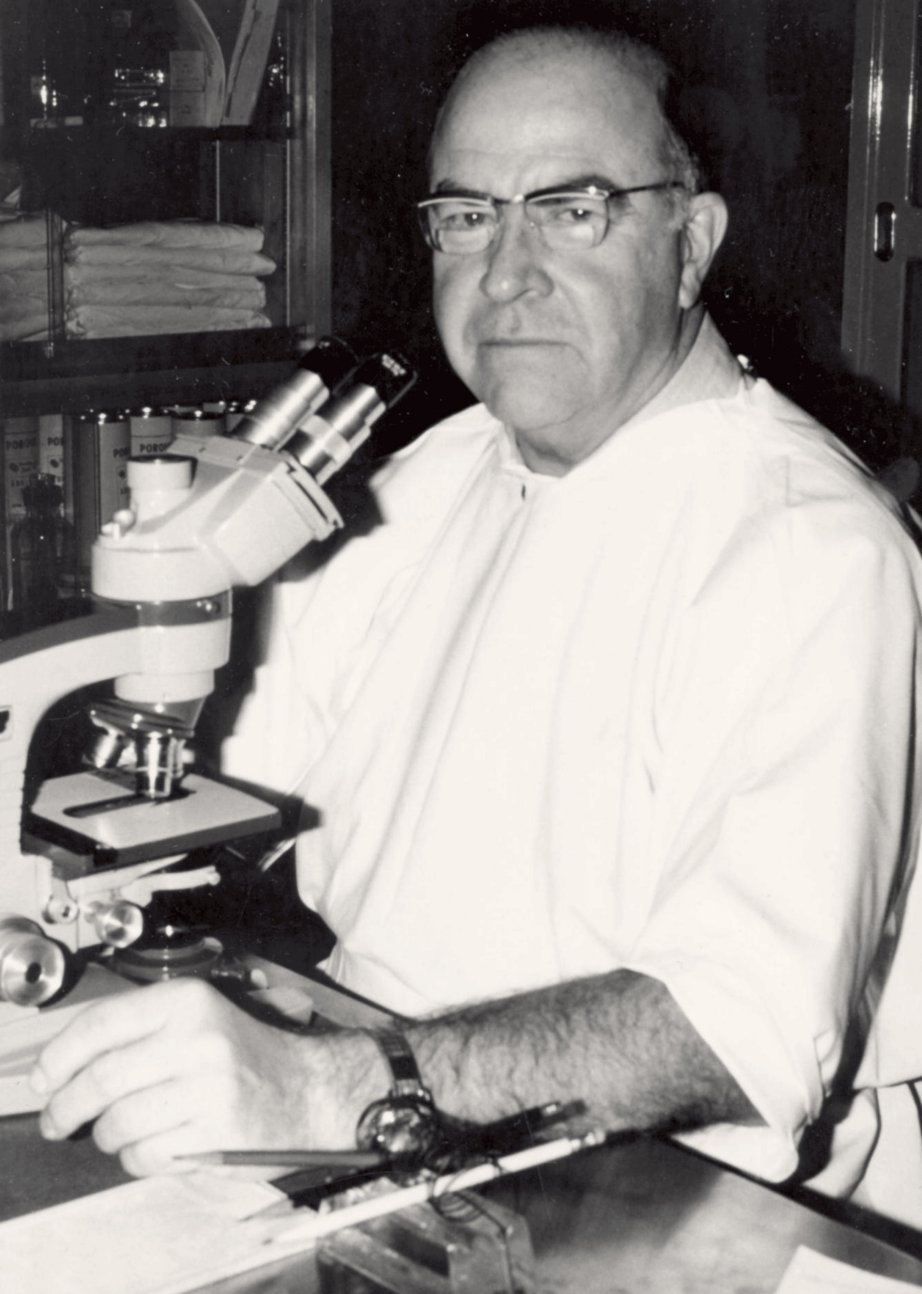
Dr. Mohs evaluating tissue microscopically Permission obtained from the University of Wisconsin Madison Archives and Record Management [4].

By 1936, after further surgical training, Dr. Mohs began performing the procedure on skin cancer patients. His original method involved applying the fixative paste, excising the fixed tissue, and then conducting a meticulous microscopic examination (Figure 2) [2,7,8]. The technique continued to evolve, leading to a significant breakthrough in 1953 when Dr. Mohs inadvertently performed a fresh tissue excision on a patient with a basal cell carcinoma of the lower eyelid. After administering local anesthesia, he excised a thin layer of fresh tissue without waiting for the fixative to set and followed the same process of sectioning and mapping and microscopic examination. The results were excellent, prompting a shift from the fixed tissue method to the fresh tissue method, which allowed for immediate tissue examination and quicker surgical decisions [9-12]. By 1963, Dr. Theodore Tromovitch expanded the use of the fresh tissue technique to various body sites, enhancing its acceptance and utility in clinical practice. A pivotal moment came in December 1970 at the annual Chemosurgery Conference when Dr. Tromovitch and Dr. Sam Stegman presented 104 cases of surgeries performed without the zinc chloride chemical fixative, reporting only four recurrences [10,11]. This marked a significant turning point, leading to the formal adoption of the fresh tissue technique as the standard. Patients benefited greatly, as surgeries and closures could be performed in one visit on the same day, allowing for immediate wound reconstruction. This advancement ultimately led to renaming the procedure from "chemosurgery" to "Mohs (micrographic) surgery."

## Review

Impact on dermatology

MMS has significantly influenced dermatology, particularly in the management of basal cell carcinoma, squamous cell carcinoma, and melanoma. Its implementation has resulted in notably higher cure rates - up to 99% for primary skin cancers and 95% for recurrent cases - especially in anatomically sensitive areas where both functional and aesthetic preservation, such as the face, eyelids, nose, and lips, are paramount [13]. The technique's success has also influenced how other cancers are approached surgically, emphasizing the importance of margin control. The focus on microscopic examination of all margins has become a key aspect of oncologic surgery, demonstrating the interdisciplinary influence of Mohs' contributions beyond dermatology alone.

Challenges and controversies

**Figure 3:**
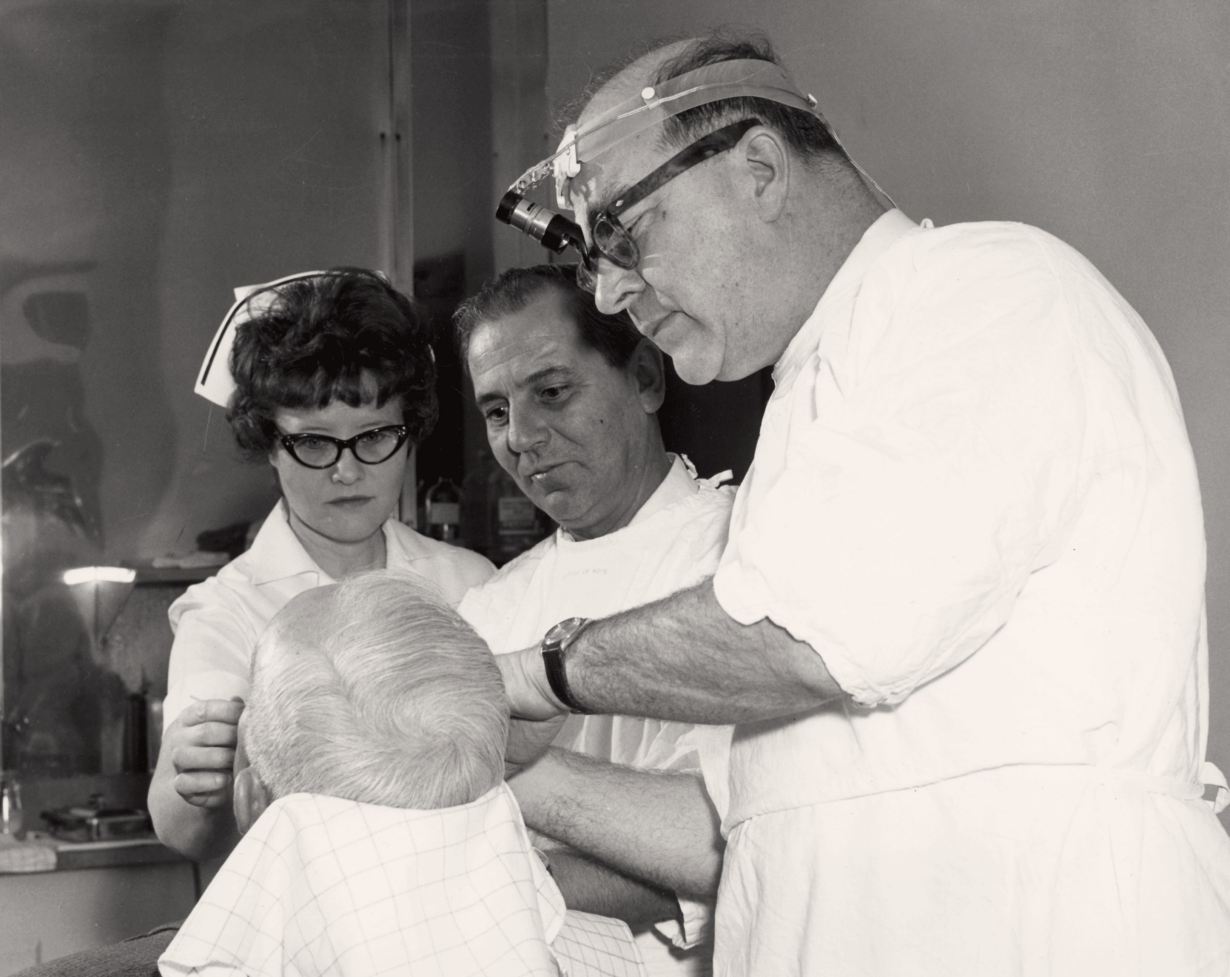
Dr. Frederic E. Mohs performing an examination or procedure Permission obtained from the University of Wisconsin Madison Archives and Record Management [4].

Despite its effectiveness, MMS initially encountered skepticism due to its complexity and the requirement for specialized training. Critics raised concerns regarding the technical demands, extended procedural time, and the necessity of specialized laboratory facilities. However, as more data emerged supporting MMS's superior outcomes - both in terms of cure rates and cosmetic results - the medical community began to embrace the technique. Over time, the undeniable benefits of MMS in terms of cure rates, tissue conservation, and reduced recurrence rates led to its widespread acceptance. Moreover, when considering modern healthcare cost structures, such as facility charges, operating room anesthesia, surgical fees, and separate pathology costs, MMS remains a highly cost-efficient procedure [1,7]. The procedure is now considered essential in dermatologic surgical practices worldwide, with increasing recognition of its value in preserving function and aesthetics, especially in complex cases (Figure 3).

Legacy and modern applications

Today, Mohs' legacy continues through the American Board of Dermatology (ABD) and the Accreditation Council for Graduate Medical Education (ACGME), with the establishment of accredited fellowships and a subspecialty board certification in this technique, and through ongoing research that seeks to refine and expand the applications of MMS. His work has not only saved countless lives but has also significantly reduced the morbidity associated with skin cancer surgery. Beyond the realm of dermatology, the principles of Mohs surgery have inspired techniques in ophthalmology, otolaryngology, and other fields where precision and tissue preservation are paramount [13]. The modern practice of Mohs surgery continues to evolve with advances in imaging, surgical tools, and adjuvant therapies, ensuring its place at the forefront of skin cancer treatment.

## Conclusions

Dr. Frederic Mohs' development of MMS stands as a testament to his vision and dedication to improving patient care. His contributions have reshaped the field of dermatologic oncology, offering hope and better outcomes for those affected by skin cancer. This paper aims to honor his legacy by detailing his life's work, its impact, and its continued relevance in the ever-evolving field of medicine. His pioneering spirit serves as an inspiration for future generations of surgeons and researchers who strive to improve patient outcomes through innovation and a commitment to excellence in medical practice. As new advancements and techniques continue to emerge, the fundamental principles established by Mohs -precision, thoroughness, and a patient-centered approach - remain cornerstones of effective skin cancer treatment.
